# Microplastics in Arctic polar waters: the first reported values of particles in surface and sub-surface samples

**DOI:** 10.1038/srep14947

**Published:** 2015-10-08

**Authors:** Amy L. Lusher, Valentina Tirelli, Ian O’Connor, Rick Officer

**Affiliations:** 1 Marine and Freshwater Research Centre, Galway-Mayo Institute of Technology, Dublin Road, Galway, Ireland; 2 OGS (Istituto Nazionale di Oceanografia e di Geofisica Sperimentale), Via A. Piccard 54, 3415, Trieste, Italy

## Abstract

Plastic, as a form of marine litter, is found in varying quantities and sizes around the globe from surface waters to deep-sea sediments. Identifying patterns of microplastic distribution will benefit an understanding of the scale of their potential effect on the environment and organisms. As sea ice extent is reducing in the Arctic, heightened shipping and fishing activity may increase marine pollution in the area. Microplastics may enter the region following ocean transport and local input, although baseline contamination measurements are still required. Here we present the first study of microplastics in Arctic waters, south and southwest of Svalbard, Norway. Microplastics were found in surface (top 16 cm) and sub-surface (6 m depth) samples using two independent techniques. Origins and pathways bringing microplastic to the Arctic remain unclear. Particle composition (95% fibres) suggests they may either result from the breakdown of larger items (transported over large distances by prevailing currents, or derived from local vessel activity), or input in sewage and wastewater from coastal areas. Concurrent observations of high zooplankton abundance suggest a high probability for marine biota to encounter microplastics and a potential for trophic interactions. Further research is required to understand the effects of microplastic-biota interaction within this productive environment.

Contamination of the world’s open oceans, enclosed seas and coastal waters by synthetic non-biodegradable material has become a high profile environmental concern^1^. Of this debris, plastic (both macro- and micro-) make up the largest quantity and can be related to increased production of anthropogenic materials and growing dependence on plastic products. World-wide production rates for 2013 were estimated at 299 million tonnes^2^. The longevity of plastics in the environment means that they can be distributed huge distances from their origin, and accumulate on remote beaches and on the seafloor^3^. Once in the ocean, mechanical and biological processes cause plastics to break down into microplastics (<5 mm)^4^,^5^. Microplastics may also enter the environment directly as granules, pellets, fibres and powders used in the production of larger plastic products, abrasive scrubs in personal care products, medicines and as a consequence of washing synthetic clothing^6^.

Microplastic monitoring tends to focus in coastal areas or in the open ocean in convergent zones and gyres^7^. Microplastics have been found worldwide, from surface waters^8^ to deep sea sediments^9^, and in freshwater systems^10^. Microplastics are difficult to mechanically remove from the ocean and their distribution and fate has still to be studied in depth. Recently, microplastic presence was reported in ice cores from remote areas of the Arctic Ocean, at levels greater than those reported for Pacific Gyre surface waters^11^. Whilst models propose the transport and a potential accumulation zone of microplastics at higher northern latitudes, including the Barents Sea^12^,^13^; field studies have not yet validated microplastic distribution within polar waters.

Polar waters support distinct and highly productive marine food webs and ecosystems^14^ which may be vulnerable to marine pollution. Prevailing ocean currents, wind currents, and migratory species can transport containments to the Arctic. In particular, the accumulation of persistent organic pollutants (POPs) has been well documented^15^. Recent quantitative analysis found the levels of plastic ingested by 87.5% of northern fulmars (*Fulmarus glacialis*) to exceed the OSPAR ecological quality objective^16^. Whilst the particular challenges posed by microplastics to polar organisms remain uncertain, interactions with microplastics have been observed for zooplankton^17^, invertebrates^18^, fish^19^, seabirds^16^, and mammals^20^. Effects of ingestion could include reduced feeding, energy depletion, injury, death or a toxicological response to contaminants associated with the plastics. As such, microplastics and marine litter have been incorporated into national and international policies, and legislation, to assess the combined risks on the environment and biota (e.g. EU Marine Strategy Framework Directive (2008/56/EC), NOAA Marine Debris Programme). Determination of the relationships between sources and sinks of microplastics will help to identify areas possibly vulnerable to microplastic accumulation.

The marine environment of the west Spitsbergen shelf has Atlantic water, Arctic water, brine and freshwater inputs^21^,^22^,^23^. Several inflowing and significant current systems transporting water, nutrients and associated biota to Arctic regions from the North Atlantic may also transport microplastics. Furthermore, as commercial activity increases in response to sea ice decline and a growing economic demand, the threat of marine pollution will also rise. This could suggest heightened levels of microplastics in the surface waters, thus increasing the likelihood of marine biota interacting with the particles. The Arctic region supports an important and diverse ecosystem from planktonic communities to marine mammals^24^. There are also several developing and important fisheries in the area^25^, which could be affected if organisms interact with marine contaminants. Considering the potential implications of microplastics^7^, there is an urgent need to assess the levels in the Arctic. This will allow for future microplastic monitoring which could lead to a risk assessment of the potential impacts of decreasing sea ice, increasing shipping and commercial activity.

This study aims to describe the distribution and abundance of polar microplastics. Two independent techniques were used to sample microplastics from surface and sub-surface waters. Water samples were collected using a manta net in the top 16 cm of surface water and sub-surface samples from the vessel’s on-board seawater pump, situated 6 m below the surface. Environmental variables including temperature, salinity, wind speed, wind direction and boat speed were collected from the vessel’s underway systems to assess their influence on the number of microplastics sampled. The present study represents the first assessment of microplastic distribution and abundance in surface and sub-surface Arctic waters south and southwest of Svalbard, Norway.

## Results

### Surface samples

**Figure 1:**
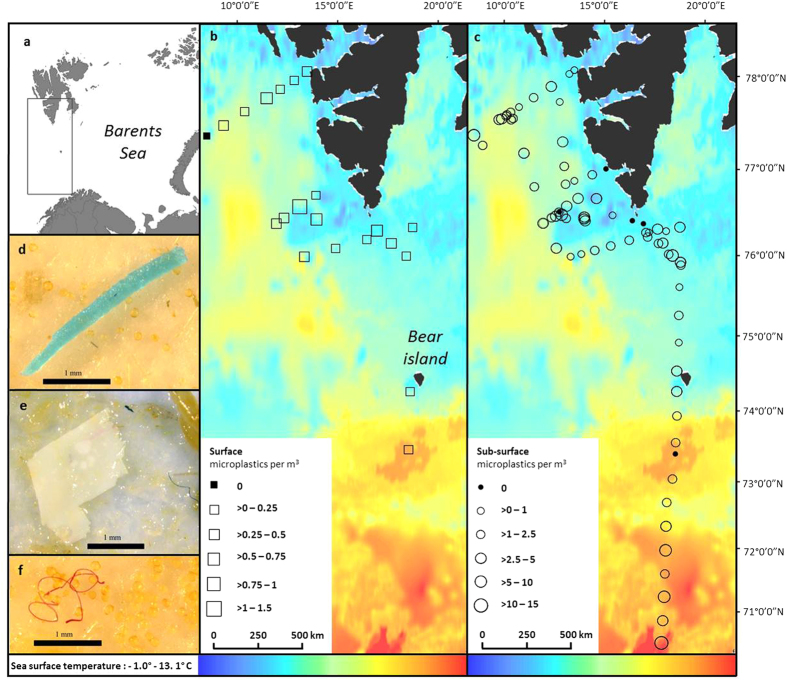
Map of sample locations during research cruise (created using ArcGIS) and example microplastic pictures. (**a**) Location of survey area, (**b**) surface sampling positions and microplastic abundance per m^3^, (**c**) sub-surface sampling positions and microplastic abundance per m^3^, (**d**) plastic fragment, (**e**) plastic film, (**f**) plastic fibre. SST source satellite data from: JPL OurOcean Project. 2010. GHRSST Level 4 G1SST Global Foundation Sea Surface Temperature Analysis. Ver. 1. PO.DAAC, CA, USA. Dataset accessed [2015-08-03] at http://dx.doi.org/10.5067/GHG1S-4FP01.

Surface samples were collected in the top 16 cm of seawater using a manta net. Microplastics were found in 20 out of 21 (95%) samples (Fig. 1b). Microplastic abundance ranged between 0 and 1.31 particles per m^3^, and averaged 0.34 (±0.31 SD) particles per m^3^. The single sample which was free from microplastics was found furthest offshore. Distances covered by the manta net tows ranged from 0.55 to 1.85 km. Mean zooplankton abundance in surface samples was 623.65 individuals per m^3^ (±838.11 SD). There was no significant correlation between zooplankton abundance and microplastic abundance (Pearson’s, *p* = 0.20).

### Sub-surface samples

Sub-surface samples were collected at a depth of 6 m using the vessel’s on-board seawater pump. Each sample consisted of 2,000 litres of sub-surface seawater filtered over a 2 hour period and the number of microplastics were standardised to m^3^. Total sub-surface sampling effort filtered 150,000 litres of seawater. Out of the 75 sub-surface samples, 93 % contained microplastics. Microplastic abundance ranged between 0 and 11.5 particles per m^3^, and averaged 2.68 (±2.95 SD) particles per m^3^ (Fig. 1c).

### Microplastic classification

A total of 665 particles were identified using visual identification methods (261 from the surface samples and 404 from the sub-surface samples). Three different types of particle were identified (fibres, fragments and films; Fig. 1d–f) with fibres being the most abundant (95%), followed by fragments (4.9%) and films (<0.1%). Black (45%) and blue (29%) were the most abundant colours. Particle size ranged between 0.25 mm and 7.71 mm with an average length of 1.93 mm (±1.22 SD). Of those particles subjected to FT-IR analysis, polymers identified included polyester (15%), polyamide (15%), polyethylene (5%), acrylic (10%), polyvinyl chloride (5%), cellulose (possibly Rayon, see discussion) (30%) and 20% of particles were of unknown origin.

### Data analysis

Average microplastic values from the surface waters 0.34 (±0.31 D) particles per m^3^ were less than those reported in sub-surface waters using the underway system 2.68 (±2.95 SD) particles per m^3^.

Whilst microplastics appear to be ubiquitous across the survey area the standardised values (microplastic count per m^3^) sampled by the manta trawl and sub-surface system were very different (GLM: *p* < 0.001, Table 1). The best fit GLM model (equation 1) of the number of microplastics per sample showed that sea surface temperature was a significant predictor of microplastic abundance (at the 0.1 significance level, Table 1).


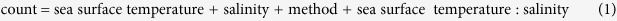


### Contamination control

Sources of contamination were mitigated through the application of established methods and controls^26^. Procedural blanks used during sampling did not indicate any sources of potential contamination.

## Discussion

Microplastics were found across the survey area in more than 90% of samples, both in the surface waters using a manta net and at 6 m depth using the vessels underway seawater pump. Microplastic abundance values in surface waters were of the same order of magnitude as those found in the North Pacific and North Atlantic, greater than the Californian current system, south and equatorial Atlantic, but less than those reported for the closed water system of the Mediterranean (Table 2). However, the variances around these values are not reported in all studies, hence precluding a determination of the potential statistical difference between them. Although the use of the sub-surface pump to collect marine debris is a relatively new method, microplastic abundance in this study (average: 2.68 ± 2.95) is not significantly higher (T-test, *t* = −0.84, *p* = 0.4) than reported using the same method in the North Atlantic (average: 2.46 microplastics per m^3 ^26). No evidence of a microplastic accumulation zone was observed. However, studies have reported increasing numbers of micro and macroplastics towards centres of ocean gyres^8^,^27^,^28^,^29^. This accumulation has been attributed to the convergence of surface currents and local wind conditions^29^.

Surface currents, wind and boat movement can cause turbulence that would be expected to redistribute particles within the water column, however, less microplastics were observed on the surface than in the sub-surface samples from this study. At present, we cannot exclude that this result could be affected by differences in the sampling methods. The two methods used cannot be directly compared as surface (manta net) and sub-surface (underway pump) sampling have some different features: the manta net filters a large volume of surface water over a short period of time and small distance (in this study up to 45 times the volume of water was sampled by the sub-surface underway pump in total) and slowly towed by the vessel; whereas the sub-surface system filters small volumes of water over larger distances, using the intake from the underway pump of the vessel. Furthermore, additional differences may arise when considering the geographical distance samples were collected over. The manta net covers a shorter distance and there would be less chance of the sampling masking the presence of possible patchiness in a smaller area compared to the sub-surface pump. Future studies with simultaneous depth sampling such as multi-nets^30^ could explain the vertical distribution of microplastics.

The number of microplastics in the study area might also be affected by the influence of water masses from adjacent areas. During the survey, marked differences in the sea-surface temperature were apparent. In general there is a cooling trend northwards and the cold Arctic Front was apparent north of Bear Island where negative anomalies in temperature and salinity marked the presence of a cyclonic gyre, or eddy, from the Barents Sea^31^. As sampling moved closer to shore, reduced salinity indicated the influence of Arctic waters and freshwater inputs from the fjords. The sub-surface samples that did not contain microplastics were reported close to the shore suggesting that unpolluted freshwater might dilute microplastic levels whereas more saline waters offshore, containing more Atlantic water, were possibly transporting microplastics from densely populated areas along the North Atlantic to the Arctic. Despite clear changes in polar fronts found throughout this survey^31^, microplastic abundances did not change significantly with salinity, however there was a marked, but not statistically significant, difference with fewer particles in colder, less saline waters. These findings could be explained by the connection between the Barents Sea and the North Atlantic. Ocean drifters released within six potential accumulation zones found that tracers from the North Atlantic gyres were advected north-eastward towards the Barents Sea, while tracers from the Barents Sea were advected south-westward towards the North Atlantic^12^. Lower numbers of microplastics were found near the cold front of the Barents Sea. Less dense Arctic water is the dominant water body in the Barents Sea and will float above the Atlantic water, suggesting that microplastic values collected here were influenced by unpolluted or diluted Arctic waters. Microplastics in Arctic water could be diluted by unpolluted freshwater rather than increased. These conclusions contrast strongly with the results of Obbard and colleagues^11^ that suggest accumulation of microplastics in the less dense, seasonally formed sea ice. The melt of ephemeral sea ice should contribute most to the less dense Arctic water. Possibly, at the time of year we sampled, the influence of unpolluted freshwater flows from the nearby Svalbard archipelago outweighed the potential microplastic input from summer sea ice melt.

Although our values are similar to those from other locations (Table 2), it is possible that the sea ice concentrates and retains microplastics, acting as a sink. As sea ice forms, it concentrates particles which are less dense than seawater and levels of microplastics in sea ice have been estimated in several orders of magnitude higher than surface waters^11^. If sea ice could act as a sink, it will be important to understand how long particles will be retained in the ice, and if/when they are released, where they will end up. Consequently, the current and forecast reduction in sea ice extent and volume^32^ could result in the release of trapped particles^11^. Inter- and intra-annual sampling will be necessary to compare to data collected in summer months, and to ascertain the role of sea ice formation and thawing processes in retaining and/or releasing entrained microplastics.

Microplastics found during our sampling were mainly fibres. This result suggests that microplastics in this area are probably from the break down products of larger plastic items such as fibres from shipping activity or fishing equipment, recreation and offshore industries (e.g. oil and gas)^6^. They have likely arrived in the Arctic after long periods at sea, and may have been transport over large distances. The input of microplastic fibres arising from the washing of textiles may also be a source^6^. However, the Arctic is not surrounded by dense urban populations, as such the input of debris from urban areas would be less likely than the transport of plastics on ocean currents, or input from shipping and commercial activity. Little is known about marine litter pollution and microplastics in Norway, although initial assessments found plastics on many Norwegian beaches including Svalbard^33^ and it is estimated that annual Norwegian emissions of microplastic are approximately 8,000 tonnes^34^. Furthermore, Arctic waters are very productive with intense fishing and ship traffic, and a large proportion of marine debris may be a result of fishing activities or loss at sea. These larger items will break down overtime to form microplastics. The direct input of microplastics to the area is at present unknown, and as such studies focusing on local and regional input, sewage and waste treatment works are required to find potential local or short-range sources. The most abundant polymers identified in this study (Rayon, polyester and polyamide, 30%, 15% and 15% respectively) were also the most abundant polymers identified in sea ice (Rayon: 54%, polyester: 21%, polyamide: 16%)^11^. Polymers identified have a range of uses, including packaging, textiles and fishing gear; as such we are unable to suggest specific sources. FT-IR reported 30% of fibres to be cellulose, however cellulose and the semi-synthetic polymer Rayon, have almost identical FT-IR spectra^26^. Without further analytical techniques, the classification must be used with caution. A review of previous studies found that the most commonly reported polymers included polyethylene and polypropylene which have low densities^35^, and are likely to float in sea water. However, as well as finding low density microplastics, we also found particles with higher densities (polyester, polyamide, acrylic, polyvinyl chloride) which are commonly used in textiles and packaging. As such particles would be more likely to sink and their presence means that some local generation of microplastics cannot be dismissed. Our observation of polymers of different densities in surface waters could also suggest that turbulence^30^, wind^36^,^37^ and storm events^38^ may redistribute particles in the water column.

The ubiquitous presence of microplastic in the surface waters of the Arctic Ocean heightens the chance that marine organisms inhabiting the same area will encounter microplastic particles. The Arctic waters support a large quantity of filter feeding organisms, from copepods, to fish and baleen whales, which could actively target or passively ingest microplastics floating in the surrounding water^7^. Previous studies have not focused on hyponeuston in the Arctic. A high abundance of zooplankton in the surface waters (our sampling collected animals present in the uppermost 16 cm of water) suggests a high probability of encounter between microplastics and fauna just beneath the surface. Within marine ecosystems zooplankton play a vital ecological role, as both primary and secondary consumers within the food web^39^. Copepods are one of the most abundant components of the zooplankton and use chemo- and mechano-receptors to detect their prey^40^. Some have the ability to distinguish between their prey (such as micro-algae or detritus) and plastic beads^41^. Effects of plastic ingestion by zooplankton observed in laboratory studies include altered feeding capacity, decreased fecundity and mortality^17^,^42^,^43^. Zooplankton collected in surface samples consisted primarily of calanoid copepods, which may actively feed on organic and synthetic particles in the surface waters. However the microplastics observed were mainly fibres, on average 1.93 mm long, and thus are unlikely to be ingested by zooplankton. Even so, further fragmentation of microplastics into nanoplastics might increase potential interaction with zooplankton that mistake plastics for prey. Secondary trophic impacts may also occur at higher trophic levels if ingested nanoplastics are transferred within the prey items of fish, birds and mammals^44^. In particular, organisms such as baleen whales that usually feed on aggregations of planktivorous fish, crustacea^45^, and cephalopods^46^ both in the water column and at the surface have a high likelihood of primary and secondary ingestion of microplastics whilst feeding. If sea ice is acting as a sink^11^ (and subsequent source of microplastics), such ecological interactions will be exacerbated by reduced sea ice extent and volume.

## Conclusion

This is the first study to show, by two independent methods, the presence and distribution of microplastics in the Arctic waters south and southwest of Svalbard, Norway, up to 78° of latitude. Microplastic found were mainly fibres (95%). This implies transport of microplastics to the Arctic possibly over long distances and periods, although the input from local sources (fishing, commercial activities and sewage) should not be overlooked. At present, we are unable to determine the source of microplastics found in this study, although it is possible that prevailing winds and surface water transport from Norway and the northeast Atlantic influence the input of microplastics to Arctic waters. We did not observe any accumulation areas but pole-ward transport pathways need to be understood to confirm whether the Arctic is acting as a sink for microplastic particles. Furthermore, the influence of local input needs to be addressed. Whilst the ecological effects of microplastic distribution in the Arctic remain uncertain, high likelihood of encounter, interaction and ingestion by marine organisms suggests there may be ecological impacts. Understanding such impacts is particularly important in biologically rich waters such as the Arctic, which support ecologically and economically valuable species and systems.

## Methods

### Sample collection

Samples were collected during an oceanographic and geophysical cruise on-board the R.V. *G. O. Sars* between June 5*th* and 15*th* 2014. Sampling started on departure from Tromsø, Norway and was carried out southwest of the Svalbard archipelago, Norway, up to the latitude of 78.07° (Fig. 1).

### Surface sample collection

Surface samples were collected using a manta trawl (0.61 m wide ×0.16 m vertical opening, 0.333 mm mesh, 3 metres long). The net was deployed from the stern of the vessel and sampled the top 10–16 cm of the water column. The net was towed in a straight line for, on average, 20 minutes at an average speed of 1.2 knots. Vessel speed was reduced to minimise the effect of vessel movement. Tow duration was maintained, however the length of the tow varied with vessel speed and surface currents, as such the volume of water filtered for each tow was not kept as a standard. A calibrated flow meter (Hydrobios) was attached to the mouth of the net to allow for calculation of the amount of water filtered. After the allocated collection time, the net was returned to the stern of the vessel where it was rinsed from the outside with a deck hose. The cod-end was removed and taken to the laboratory where it was rinsed and volume reduced, using pre-filtered water, into a sieve (200 μm). The volume reduced sample was transferred to a large collecting jar and stored in formalin (4% final concentration) for analysis. Analysis of plankton rich samples, followed an adaption of previously published procedures. Firstly, samples were gently shaken and placed in graduated cylinders to separate microplastics from zooplankton by gravity^37^. After standing for 24 hours, the organic material sank forming a concentrated deposit in the bottom of the cylinders whereas plastics floated in the overlying supernatant. The volume of the settled zooplankton was recorded before the supernatant was removed. The supernatant was filtered under vacuum onto GF/C paper (47 mm), and analysed under a Leica M205 C stereo microscope for the presence of microplastics (see **microplastic identification)**. From the remaining material, the solution was homogenised and the entire samples or replicate aliquots of 5 ml were analysed by counting at least 1000 individual zooplankton. The number of individuals found in the aliquots was multiplied for the total zooplankton volume (during this analysis the possible presence of sunk microplastic particles was investigated). A potential source of underestimation in this method is the sinking of bio-fouled or denser particles, thus excluding them from the supernatant. Any plastic found within the zooplankton replicate aliquots were added to the initial count. It is noted that this problem would only be relevant in samples which contained a large quantity of zooplankton and methods previously developed^47^ could be used to reduce underestimation of microplastic particles. To calculate the zooplankton abundance (individuals per m^3^), the zooplankton in the total volume collected was divided by the volume filtered using the estimation of the flow meter (excluding stations 20 and 22 where we used the volume calculated by distance travelled, due to error with the flow meter).

### Sub-surface sample collection

Seawater was collected from a continuous intake located on the drop keel in the centre of the vessel at a depth of 6 m. Seawater was pumped aboard using an IWAKI Magnetic Drive Pump (MDM25 160 ECFF 0221-E2) (Japan), 2.2 kW power (2 Bar vacuum) and particles were collected using previously published methodology^26^. Seawater was passed under pressure through stainless steel pipes to the deck of the vessel. Samples were collected using a hose connected directly to the seawater system. A marine grade stainless steel sieve (250 μm), in a simple covered sampling stage, filtered suspended particulate matter from a known volume of water. Calculation of the flow rate made it possible to determine the period of time required to filter a 2,000 litre volume of water (2hr). After the required elapsed time to filter 2,000 litres, any items retained on the sieve were re-suspended using filtered water and subsequently filtered under vacuum onto GF/C (47 mm diameter) filter paper. Filter papers were folded, placed in Eppendorf tubes, labelled and stored at – 20 °C. On return to the laboratory, filter papers were analysed under a stereo microscope and particles were counted and categorised following (26, see below **microplastic identification**). Environmental data (temperature, salinity, wind speed, wind direction, boat speed) were collected from the vessels underway systems.

### Contamination control

To mitigate sample contamination all glass vessels were acid washed and rinsed with pre-filtered sea water before and after use. All consumables were taken directly from packaging, and considered sterile. Samples and equipment were covered where possible to minimise periods of exposure, and rinsed with filtered water. Filters were analysed microscopically for fibres and evidence of microplastic contamination before use. Procedural blanks (absent of biological material or microplastics) were run in parallel with samples containing desiccated material, solutions or trawled material. Procedural blanks were analysed in the same way as other samples for microplastics using a stereomicroscope after filtering under pressure onto GF/C paper. Personal protective equipment, lab coats and gloves were worn at all times.

### Microplastic identification

Filter papers were visually examined under a dissecting microscope fitted with a polariser (Olympus SZX10 with a mounted Q-imaging Retiga 2000R camera and Leica M205 C), and photographs of all potential microplastics were recorded. Particles were assigned to product type categories: fibres, fragments and films (Fig. 1d,e,f). Potential microplastics were confirmed and accepted based on features such as colour: homogenously coloured, shininess or unnatural colours, if they had unnatural forms (no cellular or organic structures visible), fibres were equally thick throughout their length and had three dimensional bending. Many fibres were not uniform in thickness or colour and subsequently rejected. A subsample of microplastics (n = 30) was selected to represent the diversity of particle types found, and subjected to Fourier Transform Infrared spectroscopy (FT-IR) analysis (Perkin Elmer Spectrum Two). This confirmed the presence of cellulosic fibres (matt, non-uniform fibres) and synthetic fibres (shiny, uniform fibres). Any fibres suspected of being of a cellulosic or semi-synthetic origin were rejected from the analysis.

### Statistical Analysis

All statistical analysis was carried out in R. Data were tested for normality and homogeneity of variance. Environmental variables were averaged for the entire sample duration of each sub-surface sample (2 hr duration). Sea state could not be reliably integrated over the two hour sample period therefore wind speed which was directly recorded from the ships instruments was used. Pairwise scatter plots were used to highlight potential explanatory variables and interactions between variables. To understand the influence of environmental factors on the number of particles per sample, an integrated Generalised Linear Model (GLM) was developed for both the surface samples and the sub-surface samples. Using a stepwise process and AIC scores to include only the main effects, the GLM was analysed using an Analysis of Variance to determine which explanatory variables (Table 3) were significant predictors of the number of microplastics per m^3^. Non-significant variables were eliminated until the final model obtained (Table 1). Zooplankton data were unavailable for the sub-surface samples. Pearson’s correlation was carried out to look for a relationship between the microplastic and zooplankton abundance in manta net samples. Sea surface temperature source satellite data downloaded from: JPL OurOcean Project. 2010. GHRSST Level 4 G1SST Global Foundation Sea Surface Temperature Analysis. Ver. 1. PO.DAAC, CA, USA. Dataset accessed [2015-08-03] at http://dx.doi.org/10.5067/GHG1S-4FP01.

## Additional Information

**How to cite this article**: Lusher, A. L. *et al.* Microplastics in Arctic polar waters: the first reported values of particles in surface and sub-surface samples. *Sci. Rep.* **5**, 14947; doi: 10.1038/srep14947 (2015).

## Figures and Tables

**Table 1:** Generalised linear model (GLM) results for significant variables only based on the best fit model: (count = SST + SSS + method + SST:SSS).

Response variable	Explanatory variables	*p*	AIC
Count	SST	0.08	611.3
	SSS	0.69	
	method	<0.001	
	SST * SSS	0.13	

The response variable was the number of microplastics per sample. Gaussian distribution was assumed for the response variable. Results displayed are explanatory variables included in the final best fit model and their significance based on Analysis of Variance. Also provided is the AIC value for the model. References to the explanatory variables can be found in Table 3.

**Table 2:** Abundance of microplastics observed in manta net and neuston net studies from around the world.

Location	n/m^2^	n/m^3^	Particle abundance
Arctic waters (This study)	0.028	0.34	0–1.31/m^3^
Bering Sea^48^	.	0.004–0.19	.
North Pacific subtropical gyre^49^	.	0.116	.
North Pacific subtropical gyre^28^	0.02–0.45	.	.
South Californian current system^50^	.	0.011–0.033	0.00–3.14/m^3^
South Pacific^29^	0.027	.	0–0.40/m^2^
North Atlantic^51^	.	0.01–0.04	.
North Atlantic subtropical gyre^27^	0.0015	.	0–0.2/m^2^
Portuguese coast^52^	.	0.02–0.036	.
Equatorial Atlantic^53^	.	0.01	.
South Atlantic^54^	.	0.03	.
Mediterranean^37^	0.12	.	0–0.89/m^2^
Mediterranean^55^	0.25	.	.
Mediterranean^56^	.	0.15	0.01–0.35/m^3^

**Table 3:** List of variables assessed in the derivation of the best fitting Generalised Linear Model (GLM).

Variables		Abbreviation	Descriptor
Dependant variable	count	count	number of particles per sample
Environmental data	sea surface temperature	SST	in °C
	sea surface salinity	SSS	in %
	wind speed	WS	in metres per second
	wind direction	WD	in degrees
	distance to shore	DS	in km
Vessel information	boat speed	BS	in knots
Sample information	volume filtered	VOL	in litres
	sample length	SL	in km
Sample method	method	method	1. underway system
			2. manta net
